# Integrating transcriptomic and genomic studies for the identification of expression quantitative trait loci associated with bovine paratuberculosis

**DOI:** 10.3389/fvets.2025.1632212

**Published:** 2025-10-10

**Authors:** Gerard Badia-Bringué, Marta Alonso-Hearn

**Affiliations:** Department of Animal Health, NEIKER-Basque Institute of Agricultural Research and Development, Basque Research and Technology Alliance (BRTA), Derio, Bizkaia, Spain

**Keywords:** paratuberculosis, expression quantitative trait loci (eQTL), gene expression, transcriptomics, genomics

## Abstract

The study of the genetic basis underlying the host response to *Mycobacterium avium* subsp. *paratuberculosis* (MAP) is usually performed using genome-wide association studies (GWAS), which assess the individual association between genotyped markers, typically single-nucleotide polymorphisms (SNPs), and phenotypic traits of interest (quantitative or qualitative). However, most SNPs identified through GWAS are located in non-coding regions, making it challenging to determine their functional relevance and to link them to target genes. To date, only a limited number of cis-expression quantitative trait loci (cis-eQTLs) with effects on gene expression and susceptibility or resistance to bovine paratuberculosis (PTB) have been characterized. Cis-QTLs can influence mRNA expression by altering the level, timing, or localization of gene expression, thereby potentially contributing to variability in PTB susceptibility or resistance. This review synthesizes recent efforts to uncover the genetic architecture of resistance or susceptibility to MAP infection by integrating transcriptomic and genomic data, with a particular focus on the identification and functional interpretation of cis-eQTLs. Furthermore, we discuss the potential practical applications of validated cis-eQTLs in genomic selection programs, genetic screening assays, and CRISPR-based genome editing approaches.

## 1 Introduction

Paratuberculosis (PTB) is a multifactorial disease and is the result of the interaction between the host, *Mycobacterium avium* subsp. *paratuberculosis* (MAP), and the environment. There are several elements associated with PTB resistance and susceptibility, classified into host factors (genetics, breed, age), bacterial factors (load, strain), and environmental factors (hygienic and sanitary farm status). Age is also an important factor, since animals younger than 6 months are more susceptible (1), probably due to their still immature immune system, the higher permeability of their Peyer’s patches, and the presence of the esophageal groove, which allows contaminated milk to cross from the esophagus directly to the abomasum. Moreover, exposure to MAP is higher in calves than in adults due to ingestion of contaminated feces present in the udder. Animal species and breed are also important, as cattle are more susceptible than sheep and goats, and dairy cattle breeds are more susceptible than beef cattle breeds (2). Additionally, management practices, biosecurity, and biocontainment also are important factors that affect MAP infection (3). The minimum infective dose for calves has been estimated at 10^3^ mycobacteria, although a higher dose could infect adults (4, 5). Two distinct strains of MAP are recognized: MAP-S and MAP-C (6). MAP-S is isolated predominantly from sheep and goats, while MAP-C has a broader host range and is usually isolated from both domesticated and wild ruminant and non-ruminant species. Additionally, MAP-C is the only strain that has been isolated from human CD patients. Factors associated with resistance/susceptibility to MAP infection are summarized in Table 1. Understanding the contribution of host genetics in susceptibility and resistance to MAP infection is crucial for the development of effective control measures, treatments, or breeding strategies against MAP.

**Table 1:** Host genetics, bacterial, and environmental factors that influence MAP infection susceptibility and resistance.

Host factors	Bacterial and environmental factors
Age	MAP load
Animal species	MAP strain
Breed	Hygienic/sanitary status of the farm
Host genetics	

## 2 Expression quantitative locus (eQTL)

**Figure 1:**
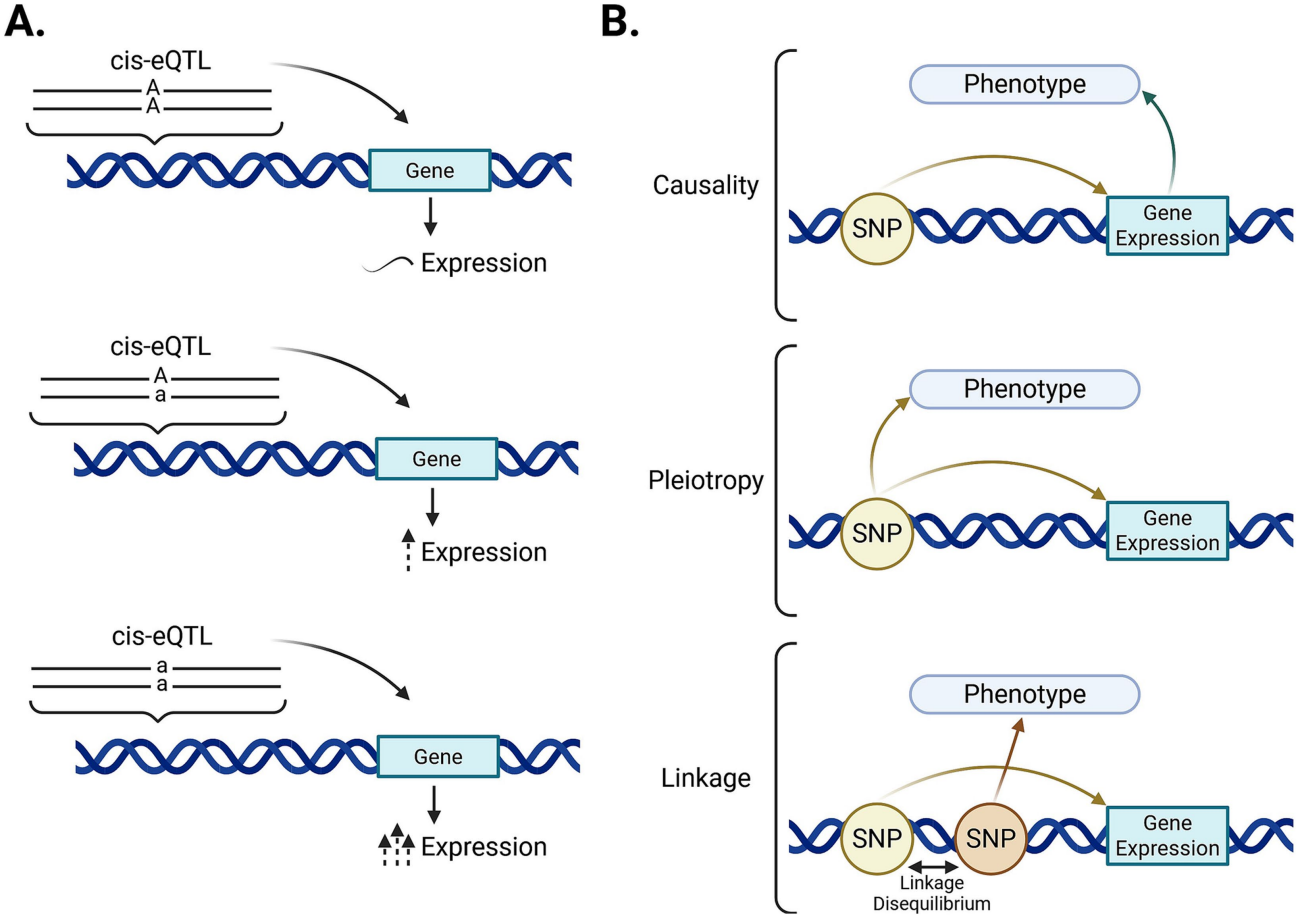
Description of cis-eQTLs associated with a specific phenotype. **(A)** Schematic representation of the relationship between a genetic variant located near a gene (cis-eQTL) and its effects on gene expression. **(B)** Associations between a genetic variant and a phenotype: causality (the variant directly affects gene expression, influencing the phenotype), pleiotropy (a single variant independently affects both gene expression and phenotype), and linkage disequilibrium (the variant is correlated with a nearby causal variant). GWAS-genome-wide association study (created with BioRender).

An eQTL is a regulatory genetic variant usually located in regulatory regions that can affect gene expression depending on the present allele (Figure 1A). eQTLs located within 1 Megabase pair of the transcription start site (TSS) of the gene they regulate are called cis-eQTLs and can modify transcription, mRNA stability, and pre-mRNA splicing (7). Therefore, cis-eQTLs that change the expression of genes associated with a phenotype will also have an influence over it. However, the mechanism by which a cis-eQTLs can influence the phenotype can be either causality, pleiotropy, or linkage disequilibrium. Figure 1B shows the direct effects that a specific allele (AA, Aa, aa) in a cis-eQTL can have on the phenotype. Under the causality model, the cis-eQTL would have a direct effect on gene expression, which would cause changes in the phenotype. Under pleiotropy, the cis-eQTL would have direct effects on both gene expression and the phenotype. In the linkage model, the cis-eQTL would not have influence on the phenotype but would be in linkage disequilibrium with the true causal variant, which would cause the two variants to be inherited together.

## 3 Cis-eQTLs identification methods

Applications of Mendelian Randomization (MR) use combined genetic-transcriptomic data for the identification of cis-eQTLs that lead to manifestations of complex diseases or disease outcomes due to genetically regulated transcriptional activity. Originally, MR was described as a method to study the causal effects of an exposure on a specific outcome using SNPs associated with exposure. Like randomized controlled trials, MR segregates the groups not based on presence or absence of exposure, but on the allelic variants in the SNPs associated with the exposure. This method is useful especially in cases where randomized controlled trials are not possible due to the nature of the exposure, it may have a high cost, or it may even be unethical. MR provides a more precise identification of the exposure-outcome relationship, as genetic variants are randomly inherited, so they should not be related to potential confounding factors that influence exposure–outcome associations (8). MR uses genetic variants to test the causative effect of an exposure (gene expression) on an outcome (phenotype of interest). However, these types of analyses need phenotypes, genotypes, and gene expression data measured in large populations, which is rarely available. Zhu et al. (9) proposed a method called summary-data-based MR (SMR), which integrates the results from GWAS analyses with data from eQTL studies in different populations to identify genes whose expression is associated with complex traits due to pleiotropy (both gene expression and the trait are affected by the same causal variant) or causality (the effect of a causal variant on the trait is mediated by gene expression)”.

## 4 Identification of PTB-associated cis-eQTLs

In the context of PTB, the phenotypes used for GWAS are ELISA, fecal bacteriological culture, and fecal PCR results. The most popular test used in GWAS is the ELISA due to its high specificity for the detection of animals with clinical signs. Several GWAS compared animals with positive ELISA test results with animals with negative ELISA test results in serum (10–15) or in milk samples (16–18). Other GWAS use results of post-mortem diagnostic tests such as bacteriological culture of tissue samples (19, 20) or histopathological analysis of gut tissues (21). Some studies combine different test results to increase specificity (10, 12, 18, 22–24). Most of the PTB-associated SNPs previously identified by GWAS are in non-coding regions of the genome, including intergenic and intronic regions, which are enriched in regulatory elements, indicating that those variants probably exert their effects through modulation of gene expression (25). Linking non-coding variants and their target genes is difficult, and only a few cis-eQTLs with effects on gene expression and PTB susceptibility or resistance have been characterized (26–29) (Table 2).

**Table 2:** Regulatory variants associated with MAP infection.

Data analysis	Number of animals and breed	Genotyped SNPs	Number of identified eQTLs /gene targets	Reference
Genomic regions of *LAMB1, DLD, WNT2, PRDM1, SOCS5, PTGER4* and *IL10* previously associated with PTB	Genotypes and expression data: 324 Holstein	8 SNPs	2 eQTLs/*WNT2, DLD*	Pauciullo et al. (30)
Three genomic regions in *SLC11A1*, *NOD2* genes previously associated with PTB	Genotypes and expression data: eight Holstein	3 SNPs	0 eQTLs	Aryngaziyev et al. (26)
A genomic region associated with positive tissue PCR and bacteriological culture	Genotypes and expression data: 221 Holstein 51 Jersey	44 SNPs 55 SNPs	2 eQTLs/*EDN2*	Kiser et al. (28)
RNA-Seq data and SNPs associated with histopathology, ELISA, tissue PCR, bacteriological culture	RNA-Seq data: 14 Holstein genotypes: 986 Holstein	54,609 SNPs	3 eQTLs/*MECOM, eEF1A2, U1*	Canive et al. (21)
RNA-Seq data and SNPs associated with the presence or absence of PTB-associated histopathological lesions	RNA-Seq data: 16 Holstein genotypes: 813 Holstein	54,609 SNPs	1 eQTLs/*CTSG*	Canive et al. (29)
RNA-Seq and GWAS data associated with the presence or absence of PTB-associated histopathological lesions	RNA-Seq data: 16 Holstein genotypes: 813 Holstein	12,377,070 SNPs	2 eQTLs/ *EGR4*, *MGC134040*	Badia-Bringué et al. (35)

WNT2-Wingless-type MMTV integration site family member 2, Dihydrolipoamide Dehydrogenase (DLD), Solute Carrier Family 11 Member 1 (SLC11A1), Nucleotide Binding Oligomerization Domain Containing 2 (NOD2), Endothelin 2 (EDN2), eukaryotic elongation factor 1-α2 (eEF1A2), U1 spliceosomal RNA, Cathepsin G (CTSG), early growth response factor 4 (EGR4), bovine neuroblastoma breakpoint family member 6-like protein isoform 2 (MGC134040).

The first analysis that integrated gene expression and genotype information to identify cis-eQTLs associated with PTB phenotypes was published by Pauciullo et al. (30). The authors selected seven genes (*LAMB1, DLD, WNT2, PRDM1, SOCS5, PTGER4*, and *IL10*) based on previously published GWAS and RNA-Sequencing (RNA-Seq) studies and validated their association with MAP infection in a population of 162 Holstein cattle positive and 162 negative for MAP infection by fecal culture and ELISA. The objective of the study was to confirm whether the selected genes and SNPs were truly associated with PTB by assessing the effects of the different alleles on the binding of transcription factors. A cis-eQTL (rs43390642: G > T) in the promoter region of *Wingless-type MMTV integration site family member 2* (*WNT2*) was associated with PTB susceptibility, suggesting a protective role of the T allele (odds ratio = 0.50). *WNT* signaling controls homeostatic self-renewal in several adult tissues, including the gut (31). In animals infected with PTB, MAP can cause granulomatous lesions in the distal part of the ileum, which may suggest a role for the *WNT* gene. The identified cis-eQTL was in linkage disequilibrium with another cis-eQTL (rs134692583: A > T) in the *dihydrolipoamide dehydrogenase* (*DLD*) previously associated with MAP infection (16). Posterior *in silico* analysis showed that the two cis-eQTLs were in binding sites for the transcription factor GR (30).

Aringaziyev et al. (26) used three genomic regions previously associated with PTB: an intron and a promoter region in the *Solute Carrier Family 11 Member 1 (SLC11A1)* gene, and an intron in the *Nucleotide Binding Oligomerization Domain Containing 2 (NOD2/CARD15)* and identified transcription factor binding sites in those regions. The effect of the alleles in those regions was tested by electrophoretic mobility shift assays (EMSA). However, EMSA did not show specific gel shifts for any allele.

To identify causal variants associated with MAP infection in Holstein and Jersey cattle, Kiser et al. (28) studied a 235-Kbp region located on chromosome 3 near the *endothelin 2* (*EDN2*) gene previously associated with MAP infection. The authors used two populations of Holstein (*N* = 221) and Jersey (*N* = 51) cattle with infectious status defined by tissue PCR and bacteriological culture. From the 44 and 53 SNPs genotyped for the Holstein and Jersey populations; 24 and 13 SNPs were found associated, respectively. After selecting the 18 most significant SNPs to perform EMSA analyses, two SNPs [rs109651404 (G/A) and rs110287192 (G/T)] located within the promoter region of the *EDN2* showed differential binding affinity for transcription factors depending on the alternate SNP alleles. The luciferase reporter assay revealed that the transcriptional activity of the *EDN2* promoter increased with the A allele for rs109651404 and the G allele for rs110287192. The role of *EDN2* in *MAP* infection may be through its interaction with macrophages, in providing intestinal mucosal immunity, or in its role in the contraction and permeability of the intestinal villi.

In 2021, Canive et al. (27) used RNA-Seq to quantify gene expression in ileocecal valve (ICV) and peripheral blood (PB) samples from 14 Holstein cattle with no lesions and with PTB-associated histopathological lesions in gut tissues. Subsequently, the associations between gene expression levels and genetic variants were analyzed by linear regression using the *Matrix eQTL* package (32). The integration of RNA-Seq data and genotype data (54,609 SNPs from a medium-density SNP chip) from a cohort of cows naturally exposed to MAP allowed the identification of 192 and 48 cis-eQTLs associated with the expression of 143 and 43 genes in PB and ICV samples, respectively. Although this study was the first to provide insights into the role of cis-eQTLs in gene transcription regulation and PTB susceptibility, it was not performed at the whole genome sequence (WGS) level. The identified cis-eQTLs were used in a GWAS analysis to identify cis-eQTLs associated with MAP infection using 839 Holstein cattle with their infectious status defined by histopathology, ELISA for MAP-antibodies detection, tissue PCR, and bacteriological culture. A total of three cis-eQTLs associated with MAP infection were identified: (1) an eQTL [rs43744169 (T/C)] associated with upregulation of the *MDS1 and EVI1 complex* (*MECOM*) was also associated with positive ELISA, PCR, and bacteriological culture results and with progression to clinical PTB; (2) an eQTL [rs110345285 (T/C)] affecting the expression of the *eukaryotic elongation factor 1-α2* (*eEF1A2*) was associated with increased optical density values of ELISA for the detection of antibodies against MAP; and (3) an eQTL [rs109859270 (C/T)] associated with the upregulation of the U1 spliceosomal RNA was also associated with an increase in the progression to clinical PTB. *MECOM* is upregulated in the presence of inflammatory stimuli, such as bacteria, and is a regulator of NF-κß (33). Genetic variants affecting the *MECOM* expression may cause an uncontrolled and aberrant inflammatory response mediated by NF-κß, which might exacerbate tissue injury in PTB-infected cattle. *eEF1A2* is a protein translation factor, and its expression is associated with protein synthesis, virus infection, and inflammatory and cancer-related processes (34). Therefore, animals with the identified variant would express higher *eEF1A2* levels and may be more susceptible to MAP infection. Finally, the variant that upregulated the U1 spliceosomal RNA may deregulate the whole splicing machinery, affecting immune processes (27).

Canive et al. (29) also investigated the effect of a cis-eQTL [rs41976219 (A/C)] affecting the expression of *Cathepsin G* (*CTSG*) in control of MAP infection and demonstrated that the heterozygous genotype was associated with higher *CTSG* levels in monocyte-derived macrophages (MDM) supernatants and lower intracellular MAP at 7 days after infection. Additionally, the homozygote minor genotype was more frequent in healthy cows than in cows with PTB-associated lesions. The CTSG is a serine protease that participates in the killing of gram-positive and gram-negative bacteria and in tissue remodeling at sites of inflammation.

In a recent study, the associations between imputed WGS genotypes and whole RNA-Seq data from PB and ICV samples of Spanish Holstein cows (*N* = 16) allowed the identification of 88 and 37 cis-eQTLs regulating the expression levels of 90 and 37 genes in PB and ICV samples, respectively (35). Next, SMR was applied to integrate the identified cis-eQTLs with GWAS results obtained from a cohort of 813 culled cattle classified according to the presence or absence of PTB-associated histopathological lesions in gut tissues to identify cis-eQTLs associated with specific histopathological lesions. After multiple testing corrections (FDR ≤ 0.05), two novel cis-eQTLs (rs383097118 and rs478694916) affecting the expression of the *early growth response* factor 4 (*EGR4*) and the *bovine neuroblastoma breakpoint family member 6-like protein* isoform *2* (*MGC134040*) were identified as pleiotropically associated with the presence of multifocal and diffuse lesions, respectively. While EGR4 acts as a brake on T-cell proliferation and cytokine production through interaction with NF-κß, MGC134040 is a target gene of NF-κß. To validate these findings, the number of EGR4-expressing cells was analyzed in paraffin-fixed gut tissues and regional lymph nodes of naturally MAP-infected Holstein Friesian cows with focal, multifocal (subclinical and clinical), and diffuse lesions (intermediate and multibacillary), and in controls without lesions by quantitative anti-EGR4 immunohistochemistry (36). Subclinical animals with multifocal lesions showed a significantly higher number of EGR4-positive cells and were sacrificed at a significantly older average age than the remaining groups (*p* < 0.001 in all cases).

## 5 Limitations

Despite their value in linking genetic variants to gene expression, current eQTL studies in livestock species face several limitations that constrain their translational utility. Most e-QTLs current studies rely on bulk RNA-Seq data from whole tissues of MAP-infected animals (37). As a result, regulatory variants acting in specific immune cell subpopulations relevant to MAP infection may remain undetected. Another limitation is that many eQTL studies in cattle are conducted with relatively small sample sizes due to the high cost and logistical complexity of transcriptomic and genomic data collection. This limits statistical power to detect variants with effects and increases the risk of false negatives. Moreover, only a limited number of eQTLs have been functionally validated using techniques such as CRISPR/Cas9, RNA interference, or reporter assays, which are essential to distinguish true causal variants from linked markers. Many cis-eQTLs studies are breed-specific, which limits the broader applicability of the identified cis-eQTLs.

## 6 Future directions

**Figure 2:**
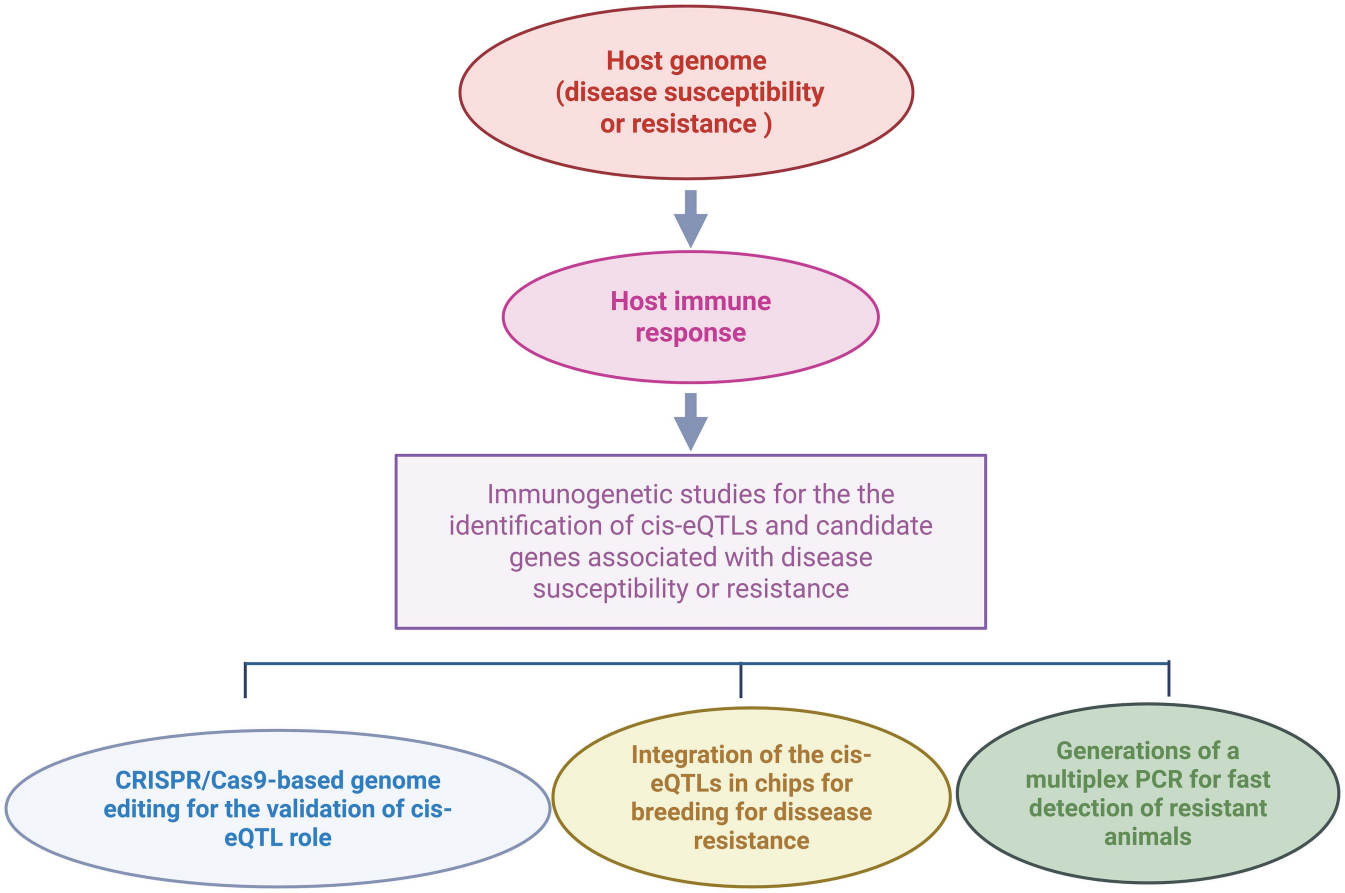
Practical applications of validated cis-eQTLs associated with resistance to MAP infection in cattle. Integrative transcriptomic-genetic approaches enable the genome-scale identification of cis-eQTLs and candidate genes related to disease resistance or susceptibility. Once validated, cis-eQTLs can be incorporated into genomic selection programs to improve disease resistance, used in multiplex PCR assays for rapid classification of animals based on genetic susceptibility or resistance, or targeted in CRISPR/Cas genome-editing strategies to modify gene expression and enhance resistance (created with BioRender).

Integrating transcriptomic and genomic studies to identify eQTLs associated with bovine PTB has significantly advanced our understanding of the genetic architecture underlying susceptibility or resistance to MAP infection in cattle. Validating the functional impact of the identified cis-eQTLs through molecular approaches such as CRISPR/Cas mediated-gene editing, reporter assays, or RNA interference is essential to elucidate how specific genetic variants modulate gene expression and contribute to host susceptibility or resistance (38, 39). Validated cis-eQTLs associated with PTB phenotypes offer multiple practical applications as illustrated in Figure 2. For instance, they could enable the development of cis-eQTLs profiling assays for the rapid and cost-effective identification of genetically susceptible or resistant animals. Furthermore, these cis-eQTLs may serve as targets for innovative PTB control strategies involving gene editing technologies. In the context of animal breeding, incorporating cis-eQTL information into genomic selection programs could enhance host resistance to MAP infection. By identifying and selectively breeding cattle with increased resistance and lower susceptibility to MAP infection, producers can improve herd health, reduce economic losses, and contribute to more sustainable and resilient livestock production systems. Although genomic selection is a medium- to long-term strategy that depends on the selective pressure and the heritability of the trait, the resulting genetic gains are cumulative and heritable across generations. Early identification of susceptible animals enables more efficient resource allocation by avoiding investment in animals at higher risk of infection. Improved productivity and disease resistance would reduce the environmental footprint of the livestock industry, supporting climate change mitigation efforts. Additionally, PTB control can alleviate trade restrictions imposed on animal and dairy products in some countries enhancing international competitiveness and market access. Ensuring animal health translates into higher quality, safer products for human consumption, thereby increasing consumer confidence. Enhanced animal health and profitability, particularly for small-scale and local farms, could also stimulate generational renewal in rural communities. The socio-economic and environmental impacts of breeding for PTB resistance are summarized in Table 3.

**Table 3:** Expecting impacts of breeding for PTB resistance.

Type of impact	Expected impact
Economic	Increase in milk production of herds affected by PTB
	Reduction in treatment costs of diseases associated with PTB
	Increase in fertility rates
	Decrease in replacement costs
	Decrease in the costs of control and prevention measures over time
Environmental	Mitigation of climate change through improved herd productivity
	More efficient use of resources (water, feed, land)
	Reduction of environmental contaminants from fewer diseased animals
Social	Sustaining the rural economy by supporting farmers and agricultural jobs
	Increased animal health and welfare
	Increased sustainability of farming systems and food production

Future research should focus on integrating eQTL data with other omics approaches, such as proteomics or metabolomics, to construct a more comprehensive, systems-level, understanding of gene regulation in MAP-infected cattle. This approach may help elucidate the molecular mechanisms influencing protein and metabolite levels in response to MAP infection. Cis-eQTLs can also regulate non-coding RNAs (ncRNAs) expression. NcRNAs, such as microRNAs (miRNAs) (37) and long non-coding RNAs (lncRNAs) (40), act as post-transcriptional regulators of gene expression during MAP infection. Although cis-eQTLs influencing ncRNAs expression have not yet been identified, integrating cis-regulatory variation with ncRNA profiles could clarify how host genetics modulates responses to MAP, enabling causal dissection of genotype, ncRNA, gene expression, and pathways relevant to MAP infection outcomes. Collectively, these insights position cis-eQTLs, miRNAs, and lncRNAs as key elements for explaining inter-animal variation in MAP susceptibility and resistance, and for developing genetics-informed breeding strategies for PTB control. Emerging technologies such as single-cell RNA-Seq also offer the potential to dissect eQTLs effects at the cellular level, thereby revealing gene regulatory networks specific to distinct immune cell populations.

## 7 Conclusion

This review article highlights a critical area at the intersection of transcriptomics, genetics, and infectious disease research. It emphasizes the value of integrating transcriptomic and genetic data to identify cis-eQTLs that influence host immune responses to MAP infection. By summarizing current findings from MAP-infected cattle, this review provides valuable insights for researchers in comparative immunology, immunogenetics, veterinary science, livestock genomics, and cattle breeding. Moreover, it highlights regulatory cis-eQTLs and their putative target genes for future functional studies.

## References

[1] Mortier RA Barkema HW Bystrom JM Illanes O Orsel K Wolf R. Evaluation of age-dependent susceptibility in calves infected with two doses of *Mycobacterium avium* subspecies *paratuberculosis* using pathology and tissue culture. Vet Res. (2013) 44:94. doi: 10.1186/1297-9716-44-94, PMID: 24099491 PMC4021370

[2] Pence M Baldwin C Black CC. The seroprevalence of Johne’s disease in Georgia beef and dairy cull cattle. J Vet Diagn Invest. (2003) 15:475–7. doi: 10.1177/104063870301500514, PMID: 14535551

[3] Donat K Schmidt M Köhler H Sauter-Louis C. Management of the calving pen is a crucial factor for paratuberculosis control in large dairy herds. J Dairy Sci. (2016) 99:3744–52. doi: 10.3168/jds.2015-10625, PMID: 26947285

[4] Gilmour NJL Nisbet DI Brotherston JG. Experimental oral infection of calves with Mycobacterium johnei. J Comp Pathol. (1965) 75:281–6. doi: 10.1016/0021-9975(65)90033-2, PMID: 5322919

[5] Windsor PA Whittington RJ. Evidence for age susceptibility of cattle to Johne’s disease. Vet J. (2010) 184:37–44. doi: 10.1016/j.tvjl.2009.01.007, PMID: 19246220

[6] Stevenson K Ahlstrom C. Comparative genomics and genomic epidemiology of *Mycobacterium avium* subsp. paratuberculosis strains In: Paratuberculosis: Organism, disease, control. CAB International Oxfordshire, UK. (2020). 76–91.

[7] Westra H-J Franke L. From genome to function by studying eQTLs. Biochim Biophys Acta. (2014) 1842:1896–902. doi: 10.1016/j.bbadis.2014.04.024, PMID: 24798236

[8] Richmond RC Davey Smith G. Mendelian randomization: concepts and scope. Cold Spring Harb Perspect Med. (2022) 12:a040501. doi: 10.1101/cshperspect.a040501, PMID: 34426474 PMC8725623

[9] Zhu Z Zhang F Hu H Bakshi A Robinson MR Powell JE. Integration of summary data from GWAS and eQTL studies predicts complex trait gene targets. Nat Genet. (2016) 48:481–7. doi: 10.1038/ng.3538, PMID: 27019110

[10] Minozzi G Buggiotti L Stella A Strozzi F Luini M Williams JL. Genetic loci involved in antibody response to *Mycobacterium avium* ssp. *paratuberculosis* in cattle. PLoS One. (2010) 5:e11117. doi: 10.1371/journal.pone.0011117, PMID: 20559561 PMC2886106

[11] Pant SD Schenkel FS Verschoor CP You Q Kelton DF Moore SS. A principal component regression based genome wide analysis approach reveals the presence of a novel QTL on BTA7 for MAP resistance in Holstein cattle. Genomics. (2010) 95:176–82. doi: 10.1016/j.ygeno.2010.01.001, PMID: 20060464

[12] Kirkpatrick BW Shi X Shook GE Collins MT. Whole-genome association analysis of susceptibility to paratuberculosis in Holstein cattle. Anim Genet. (2011) 42:149–60. doi: 10.1111/j.1365-2052.2010.02097.x, PMID: 20618184

[13] Gao Y Jiang J Yang S Cao J Han B Wang Y. Genome-wide association study of *Mycobacterium avium* subspecies Paratuberculosis infection in Chinese Holstein. BMC Genomics. (2018) 19:972. doi: 10.1186/s12864-018-5385-3, PMID: 30591025 PMC6307165

[14] Mallikarjunappa S Sargolzaei M Brito LF Meade KG Karrow NA Pant SD. Short communication: uncovering quantitative trait loci associated with resistance to *Mycobacterium avium* ssp. paratuberculosis infection in Holstein cattle using a high-density single nucleotide polymorphism panel. J Dairy Sci. (2018) 101:7280–6. doi: 10.3168/jds.2018-14388, PMID: 29753465

[15] McGovern SP Purfield DC Ring SC Carthy TR Graham DA Berry DP. Candidate genes associated with the heritable humoral response to *Mycobacterium avium* ssp. *paratuberculosis* in dairy cows have factors in common with gastrointestinal diseases in humans. J Dairy Sci. (2019) 102:4249–63. doi: 10.3168/jds.2018-15906, PMID: 30852025

[16] van Hulzen KJE Schopen GCB van Arendonk JAM Nielen M Koets AP Schrooten C. Genome-wide association study to identify chromosomal regions associated with antibody response to *Mycobacterium avium* subspecies paratuberculosis in milk of Dutch Holstein-Friesians. J Dairy Sci. (2012) 95:2740–8. doi: 10.3168/jds.2011-5005, PMID: 22541504

[17] Brito LF Mallikarjunappa S Sargolzaei M Koeck A Chesnais J Schenkel FS. The genetic architecture of milk ELISA scores as an indicator of Johne’s disease (paratuberculosis) in dairy cattle. J Dairy Sci. (2018) 101:10062–75. doi: 10.3168/jds.2017-14250, PMID: 30219422

[18] Kirkpatrick BW Cooke ME Frie M Sporer KRB Lett B Wells SJ. Genome-wide association analysis for susceptibility to infection by *Mycobacterium avium* ssp. paratuberculosis in US Holsteins. J Dairy Sci. (2022) 105:4301–13. doi: 10.3168/jds.2021-21276, PMID: 35307176

[19] Kiser JN White SN Johnson KA Hoff JL Taylor JF Neibergs HL. Identification of loci associated with susceptibility to *Mycobacterium avium* subspecies *paratuberculosis* (map) tissue infection in cattle. J Anim Sci. (2017) 95:1080–91. doi: 10.2527/jas2016.1152 28380509

[20] Settles M Zanella R McKay SD Schnabel RD Taylor JF Whitlock R. A whole genome association analysis identifies loci associated with *Mycobacterium avium* subsp. *paratuberculosis* infection status in US holstein cattle. Anim Genet. (2009) 40:655–62. doi: 10.1111/j.1365-2052.2009.01896.x, PMID: 19422364

[21] Canive M Badia-Bringué G Vázquez P González-Recio O Fernández A Garrido JM. Identification of loci associated with pathological outcomes in Holstein cattle infected with *Mycobacterium avium* subsp. paratuberculosis using whole-genome sequence data. Sci Rep. (2021) 11:20177. doi: 10.1038/s41598-021-99672-4, PMID: 34635747 PMC8505495

[22] Alpay F Zare Y Kamalludin MH Huang X Shi X Shook GE. Genome-wide association study of susceptibility to infection by *Mycobacterium avium* subspecies paratuberculosis in Holstein cattle. PLoS One. (2014) 9:e111704. doi: 10.1371/journal.pone.0111704, PMID: 25473852 PMC4256300

[23] Zare Y Shook GE Collins MT Kirkpatrick BW. Genome-wide association analysis and genomic prediction of *Mycobacterium avium* subspecies paratuberculosis infection in US Jersey cattle. PLoS One. (2014) 9:e88380. doi: 10.1371/journal.pone.0088380, PMID: 24523889 PMC3921184

[24] Canive M González-Recio O Fernández A Vázquez P Badia-Bringué G Lavín JL. Identification of loci associated with susceptibility to *Mycobacterium avium* subsp. paratuberculosis infection in Holstein cattle using combinations of diagnostic tests and imputed whole-genome sequence data. PLoS One. (2021) 16:e0256091. doi: 10.1371/journal.pone.0256091, PMID: 34449805 PMC8396740

[25] Farh KK-H Marson A Zhu J Kleinewietfeld M Housley WJ Beik S. Genetic and epigenetic fine mapping of causal autoimmune disease variants. Nature. (2015) 518:337–43. doi: 10.1038/nature13835, PMID: 25363779 PMC4336207

[26] Aryngaziyev B Beltramo C Dondo A Karymsakov T Varello K Goria M. Polymorphisms associated to bovine paratuberculosis: investigation of their role in DNA-protein interactions and transcriptional regulation. Vet Ital. (2020) 56:109–14. doi: 10.12834/VetIt.2325.13205.1, PMID: 32761582

[27] Canive M Fernandez-Jimenez N Casais R Vázquez P Lavín JL Bilbao JR. Identification of loci associated with susceptibility to bovine paratuberculosis and with the dysregulation of the MECOM, eEF1A2, and U1 spliceosomal RNA expression. Sci Rep. (2021) 11:313. doi: 10.1038/s41598-020-79619-x, PMID: 33432064 PMC7801378

[28] Kiser JN Wang Z Zanella R Scraggs E Neupane M Cantrell B. Functional variants surrounding endothelin 2 are associated with *Mycobacterium avium* subspecies paratuberculosis infection. Front Vet Sci. (2021) 8:625323. doi: 10.3389/fvets.2021.625323, PMID: 34026885 PMC8131860

[29] Canive M Badia-Bringué G Alonso-Hearn M. The upregulation of cathepsin G is associated with resistance to bovine paratuberculosis. Animals. (2022) 12:1–12. doi: 10.3390/ani12213038 PMC9655680 36359162

[30] Pauciullo A Küpper J Brandt H Donat K Iannuzzi L Erhardt G. *Wingless-type MMTV integration site family member 2* (*WNT2*) gene is associated with resistance to MAP in faecal culture and antibody response in Holstein cattle. Anim Genet. (2015) 46:122–32. doi: 10.1111/age.12261, PMID: 25643727

[31] Fevr T Robine S Louvard D Huelsken J. Wnt/β-catenin is essential for intestinal homeostasis and maintenance of intestinal stem cells. Mol Cell Biol. (2007) 27:7551–9. doi: 10.1128/MCB.01034-07, PMID: 17785439 PMC2169070

[32] Shabalin AA. Matrix eQTL: ultra fast eQTL analysis via large matrix operations. Bioinformatics. (2012) 28:1353–8. doi: 10.1093/bioinformatics/bts163, PMID: 22492648 PMC3348564

[33] Xu X Woo C-H Steere RR Lee BC Huang Y Wu J. EVI1 acts as an inducible negative-feedback regulator of NF-κB by inhibiting p65 acetylation. J Immunol. (2012) 188:6371–80. doi: 10.4049/jimmunol.1103527, PMID: 22581859 PMC3370108

[34] Lee M Surh Y. eEF1A2 as a putative oncogene. Ann N Y Acad Sci. (2009) 1171:87–93. doi: 10.1111/j.1749-6632.2009.04909.x, PMID: 19723040

[35] Badia-Bringué G Canive M Fernandez-Jimenez N Lavín JL Casais R Blanco-Vázquez C. Summary-data based Mendelian randomization identifies gene expression regulatory polymorphisms associated with bovine paratuberculosis by modulation of the nuclear factor kappa β (NF-κß)-mediated inflammatory response. BMC Genomics. (2023) 24:605. doi: 10.1186/s12864-023-09710-w, PMID: 37821814 PMC10568764

[36] Navarro León AI Alonso-Hearn M Muñoz M Iglesias N Badia-Bringué G Iglesias T. Early growth response factor 4 (EGR4) expression in gut tissues and regional lymph nodes of cattle with different types of Paratuberculosis-associated lesions: potential role of EGR4 in resilience to Paratuberculosis. Animals. (2025) 15:1012. doi: 10.3390/ani15071012, PMID: 40218405 PMC11988129

[37] Badia-Bringué G Alonso-Hearn M. RNA-sequencing studies suggest that microRNAs and alternative splicing of pre-mRNAs modulate immune and inflammatory responses in Holstein cattle infected with Mycobacterium avium subsp. paratuberculosis. Front Immunol (2025) 16:1–12.:1597736 doi: 10.3389/fimmu.2025.1597736, PMID: 40636120 PMC12237680

[38] Mallikarjunappa S Brito LF Pant SD Schenkel FS Meade KG Karrow NA. Johne’s disease in dairy cattle: an immunogenetic perspective. Front Vet Sci. (2021) 8:1–19. doi: 10.3389/fvets.2021.718987 PMC8426623 34513975

[39] Jabbar A Zulfiqar F Mahnoor M Mushtaq N Zaman MH din ASU. Advances and perspectives in the application of CRISPR-Cas9 in livestock. Mol Biotechnol. (2021) 63:757–67. doi: 10.1007/s12033-021-00347-2, PMID: 34041717

[40] Badia-Bringué G Asselstine V Cánovas Á Alonso-Hearn M. Genome-wide long non-coding RNA expression profile and its regulatory role in the ileocecal valve from *Mycobacterium avium* subsp. *paratuberculosis*-infected cattle. Front Vet Sci. (2025) 12:1601267. doi: 10.3389/fvets.2025.1601267, PMID: 40538732 PMC12176553

